# Mast cell leukemia with complex genomic alterations in an elderly patient with prior hematologic and solid malignancies: a case report

**DOI:** 10.1097/MS9.0000000000005426

**Published:** 2026-07-30

**Authors:** Angad Tiwari, Ashish Sharma, Jhalak Agrohi, Khabab Abbasher Hussien Mohamed Ahmed, Ritika Vankina, Sukhmani Sidhu

**Affiliations:** a Department of Internal Medicine, Maharani Laxmi Bai Medical College, Jhansi, Uttar Pradesh, India; b University of Connecticut School of Medicine, Hartford, CT, USA; c John H. Stroger Hospital of Cook County, Chicago, IL, USA; d Faculty of Medicine, University of Khartoum, Khartoum, Sudan; e Department of Pathophysiology, St. George’s University, St. George’s, Grenada; f Department of Hematology and Oncology, University of Connecticut School of Medicine, Hartford, CT, USA; g University of Connecticut, Department of Internal Medicine, Farmington, CT, USA

**Keywords:** case report, diffuse large B-cell lymphoma, genetic alterations, mast cell leukemia, prostate adenocarcinoma

## Abstract

**Introduction:**

Mast cell leukemia (MCL) is the rarest and most aggressive variant of systemic mastocytosis (approximately 1% of cases), with a median survival of under 2 years. Diagnosis requires ≥20% atypical mast cells in the marrow aspirate, and the disease frequently overlaps with myeloid neoplasms.

**Case presentation:**

An 86-year-old man with paranasal sinus diffuse large B-cell lymphoma in remission since 2017 after R-CHOP and methotrexate, and prostate adenocarcinoma treated in 2019, presented with acute pancytopenia, presumed to represent lymphoma relapse. Serum tryptase exceeded 11 999 ng/mL; the aspirate showed 20% pleomorphic mast cells (CD117+, weak CD25, CD2−), confirming aleukemic MCL. Formal CMML criteria could not be confirmed due to the unavailability of monocyte differential data; however, the findings raised suspicion for an associated myeloid neoplasm, with SM with an associated hematological neoplasm remaining an alternative classification. Karyotyping was normal; next-generation sequencing revealed pathogenic variants in TP53, RB1, DAXX, ASXL1, TET2, and SRSF2, and a rare extracellular-domain KIT p.D419del. He declined inpatient midostaurin, deteriorated rapidly, and died 2 weeks later.

**Clinical discussion:**

This case illustrates a therapy-related MCL (plausible but unconfirmed given the non-leukemogenic profile of methotrexate and the focal nature of prostate stereotactic body radiation therapy) with a suspected associated myeloid neoplasm and complex pathogenic mutations. The KIT p.D419del extracellular domain variant is a rare non-D816V mutation; the canonical D816V was not detected on NGS, though the presence of a low-variant allele fraction D816V cannot be fully excluded due to assay sensitivity. Despite midostaurin, the disease remained aggressive.

**Conclusion:**

Persistent unexplained cytopenias warrant heightened suspicion of MCL, and comprehensive genomic profiling clarifies diagnosis, distinguishes overlapping myeloid disease, and informs prognosis in this aggressive, refractory neoplasm.

## Introduction

Mast cell leukemia (MCL), first described by Joachim in 1906, is the least common form of systemic mastocytosis (SM), accounting for approximately 1% of all SM cases^[^1–3^]^. In most cases, organ damage and a poor prognosis are associated with a median overall survival (OS) of 1.6 years in patients with MCL. In the bone marrow (BM) and other organs, it is characterized by the leukemic proliferation of predominantly immature mast cells (MCs)^[^1,4^]^. It often coexists with other myeloid neoplasms, such as CMML and myelodysplastic syndromes (MDS)^[^3^]^. Patients with MCL often have elevated blood tryptase levels, cytopenias, and organomegaly^[^2^]^.

HIGHLIGHTS

- Aleukemic mast cell leukemia developed in an elderly patient with prior hematologic and solid malignancies.
- Genomic profiling revealed a rare KIT p.D419del mutation alongside alterations in TP53, RB1, DAXX, ASXL1, TET2, and SRSF2.
- Molecular findings raised suspicion of overlapping myeloid neoplasms and refined the diagnostic classification.
- The case underscores the value of comprehensive genomic testing for prognostication and therapeutic decision-making in mast cell leukemia.

To diagnose MCL, BM smears must show at least 20% atypical, immature MCs, and the patient must also meet the diagnostic criteria for SM^[^5^]^. Due to the uncommon nature of this condition, little is known about its causes and potential therapies. Although there are few options, several therapies for SM, including corticosteroids, chemotherapy, BM transplants, targeted therapies, and stem cell transplants, may be considered lines of treatment for MCL. The efficacy of these treatments for MCL is still being investigated^[^2^]^.

This case illustrates the difficult task of managing a complicated clinical picture and the complex interplay of numerous hematologic malignancies in the process of making a differential diagnosis for a patient exhibiting unexplained cytopenias. It also highlights the extent to which further study is required in order to comprehend and manage this condition.

This report adheres to the TITAN Guidelines 2025 for artificial intelligence declaration in scientific writing^[^6^]^.

## Case

An 86-year-old male with a medical history of intermediate-risk prostate adenocarcinoma and stage IV diffuse large B-cell lymphoma (DLBCL) diagnosed in September 2017 presented to our facility with acute pancytopenia. Prior to December 2017, the patient received therapy for central nervous system prophylaxis with R-CHOP and methotrexate; subsequent surveillance revealed no evidence of disease.

Initial laboratory tests showed a hemoglobin of 9.5 g/dL, a hematocrit of 29.4%, a white blood cell count of 1.03 × 10^3^/µL, and a platelet count of 19 000/µL. Thrombocytopenia with no immature cells was observed on the peripheral smear, consistent with the aleukemic variant of MCL.

The diagnosis was supported by a serum tryptase level of >11 999 ng/mL. This value reflects the upper limit of quantification of the commercial ImmunoCAP Tryptase assay; values exceeding this threshold have been documented in severe MC activation and MCL and should not be interpreted as transcription errors.

BM aspirate differential count revealed pleomorphic MCs comprising 20% of the nucleated cells. This figure was derived from the BM aspirate differential count of 500 nucleated cells, as required by the World Health Organization (WHO) 2022 diagnostic criteria for MCL, and is distinct from the biopsy-based cellularity estimate or immunohistochemistry-based approximation. The diagnosis of MCL rather than aggressive SM was based on this aspirate-derived threshold. Immunophenotyping showed positivity for CD117 and CD33, with variable to weak CD25 expression and negativity for CD2 and CD34.

Conventional cytogenetic analysis showed no clonal abnormalities, most likely representing a chemotherapeutic effect. Next-generation sequencing confirmed clonal disease with the following pathogenic mutations and variant allele fractions (VAFs): TP53 (50%), RB1 (40%), DAXX (40.2%), KIT p.D419del (38%), ASXL1 (34.9%), TET2 splice-region variant/loss-of-function (12, 9, and 2.4%), and SRSF2 (4.8%). These results are summarized in Table 1.

**Table 1:** Next-generation sequencing results and clinical correlates.

Gene	VAF (%)	Classification	Functional significance	Clinical relevance
TP53	50	Pathogenic	Tumor suppressor; possible LOH/biallelic inactivation	Poor prognosis; therapy-related risk
RB1	40	Pathogenic	Cell cycle regulation; early MDS-to-AML transition marker	Disease aggressiveness
DAXX	40.2	Pathogenic	Chromatin remodeling; associated with prostate cancer (high Gleason)	Uncertain in MCL
KIT p.D419del	38	Likely pathogenic	Extracellular domain (exon 8); rare non-D816V variant; activating mechanism uncertain	Midostaurin used; avapritinib not approved for non-D816V
ASXL1	34.9	Pathogenic	Epigenetic regulator; CMML/MDS associated	Inferior OS in advanced SM
TET2 (x3)	12, 9, and 2.4	Pathogenic (LoF)	DNA demethylation; multiple variants suggesting biallelic involvement	CMML-associated; inferior OS
SRSF2	4.8	Pathogenic	RNA splicing; subclonal event	CMML-associated; late clonal event

Summary of NGS-identified pathogenic mutations with VAF, functional significance, and clinical relevance. Orange highlighting denotes the novel KIT p.D419del extracellular-domain variant.

LOH, loss of heterozygosity; LoF, loss of function; SM-AHN; VAF, variant allele fraction.

The presentation was most consistent with aleukemic MCL, with features raising suspicion for an associated myeloid neoplasm. Specifically, the combination of moderate dysplasia and a mutation profile including ASXL1, TET2, and SRSF2 raised concern for an overlapping CMML-like process; however, the formal 2022 WHO CMML diagnostic criteria, including persistent monocytosis and the monocyte percentage on the differential, could not be confirmed due to the unavailability of complete monocyte differential data in the medical record. SM-AHN, therefore, remains a plausible alternative classification and cannot be excluded.

Midostaurin was offered as targeted therapy, given the presence of a KIT mutation. The patient declined formal inpatient treatment and was managed with supportive care. His clinical condition deteriorated rapidly within days of presentation, and he passed away approximately 2 weeks after the index BM biopsy. Avapritinib, cladribine, and hydroxyurea were considered but not administered, given the pace of clinical deterioration and the patient's goals of care. No imaging data or BM fibrosis grading was available in the medical record for this case review.

## Discussion

An abnormal proliferation and accumulation of MCs and their CD34+ progenitor cells is known as mastocytosis, a neoplastic condition. MCs in different organ systems or tissues, most frequently the skin and BM, exhibit clonal proliferation as a characteristic of this pathology. It can also affect the liver, spleen, lymph nodes, and gastrointestinal system^[^7^]^. The WHO classifies mastocytosis into three distinct entities: cutaneous mastocytosis, SM, and MC sarcoma. Aggressive forms include aggressive SM, SM-AHN, and MCL, which together constitute advanced SM^[^7^]^.

This case highlights the diagnostic challenges associated with MCL – the rarest subtype of SM and invariably associated with the worst prognosis – in a patient with prior DLBCL and prostate adenocarcinoma^[^8^]^. MCL is diagnosed based on BM biopsy findings indicating infiltration by immature MCs and on a BM aspirate smear revealing ≥20% atypical MCs. This case represents the aleukemic variant, given the absence of immature cells on peripheral smear.

In this case, the patient’s clinical profile, with immature MCs comprising more than 20% of the BM aspirate, alongside acute pancytopenia, was key to the diagnosis of MCL. In SM, aberrant MCs are identified using immunophenotyping. The BM showed pleomorphic MCs positive for CD117 and CD33, with weak/variable CD25 expression, and negative for CD2 and CD34. The weak CD25 expression in this case deserves mention: while CD25 is a sensitive marker for neoplastic MCs, approximately one-third of MCL patients lack CD2 and CD25 expression, and their absence should not preclude diagnosis when other criteria are met^[^9^]^. The negativity for CD34 argues against a significant myeloid blast component, helping to distinguish this from transformation to acute myeloid leukemia. Tryptase, CD117, CD25, and CD2 are important indicators for recognizing atypical MCs in SM^[^9^]^. Elevated tryptase provides further evidence for MCL in this case.

More than 80% of SM cases harbor a somatic mutation in *KIT* at codon 816, most commonly *KIT* p.D816V^[^7^]^. In MCL specifically, D816V prevalence ranges between 46 and 68%^[^7^]^. The KIT p.D419del variant identified in this case is located in the extracellular domain (exon 8), distinct from the canonical activation-loop mutation at D816V (exon 17). This extracellular-domain location is rare in mastocytosis, and while limited functional data exist, KIT exon 8 mutations have been reported in both SM and gastrointestinal stromal tumors, with the 38% VAF strongly suggesting a clonal driver event. In a study by Kennedy *et al*^[^3^]^ examining prognostic factors in patients with MCL, KIT D816V positivity was associated with better OS compared with those with alternative KIT mutations or absence of KIT mutations. Importantly, canonical KIT D816V was not detected by the NGS panel used in this case. However, we acknowledge that standard NGS may not reliably detect low-VAF D816V alleles; a highly sensitive assay such as allele-specific PCR or droplet digital PCR was not performed. This represents a limitation of our molecular workup and is relevant because D816V status influences eligibility for avapritinib, which is specifically approved for KIT D816V-positive advanced SM.

Recent studies indicate that neoplastic cells in >60% of all patients with advanced SM demonstrate somatic variants in genes other than *KIT*. Among these, TET2, SRSF2, ASXL1, CBL, and RUNX1 are the most frequently affected^[^10^]^. These were associated with inferior OS in the Kennedy *et al*^[^3^]^ registry study, which also reported inferior OS for leukemic versus aleukemic MCL patients (0.4 vs. 1.9 years).

The TP53 mutation found in this patient (VAF of 50%) likely represents an early truncal event and raises the possibility of biallelic inactivation or loss of heterozygosity (LOH). A VAF of approximately 50% in a clonal myeloid neoplasm is suspicious for LOH, which can occur via deletion of the wild-type allele, copy-neutral LOH, or mitotic recombination. Biallelic TP53 inactivation confers a particularly poor prognosis in myeloid neoplasms and may have contributed to the fulminant course observed in this case^[^11^]^. A few cases of MCL with TP53 mutations have been reported, all with similarly poor prognoses ^[^11^]^.

The range of observed VAFs among different mutations (4.8–50%) likely reflects the heterogeneous clonal architecture of the disease. Higher VAF alterations, including TP53 (50%), RB1 (40%), DAXX (40.2%), and KIT p.D419del (38%), likely represent clonal drivers of the common ancestral clone. Lower VAF alterations, particularly SRSF2 (4.8%) and the smallest TET2 variant (2.4%), may represent later subclonal events, consistent with ordered clonal hierarchies identified in CMML and other myeloid malignancies^[^12,13^]^.

A study examining the role of the retinoblastoma gene (RB1), which encodes a tumor suppressor protein, in hematological malignancies stated that changes in RB1 are observed early in the transition from MDS to acute leukemia. DAXX gene mutations, while not well-studied in MCL, have been reported in prostate cancer patients with a high Gleason score or metastatic disease^[^14,15^]^.

The question of therapy-related pathogenesis in this case warrants careful framing. The patient had received R-CHOP (which includes cyclophosphamide and doxorubicin – alkylating and topoisomerase II–targeting agents, respectively) and intrathecal and systemic methotrexate approximately 5–6 years prior to MCL presentation. The latency period is compatible with therapy-related myeloid neoplasm, classically defined as occurring 5–10 years after alkylating agent exposure. However, several considerations temper a definitive therapy-related attribution: methotrexate is an antimetabolite and is not classically leukemogenic; stereotactic body radiation therapy to the prostate delivers focal radiation to a field unlikely to encompass substantial marrow space; and a TP53 mutation at 50% VAF may represent age-related clonal hematopoiesis of indeterminate potential (CHIP) predating cytotoxic therapy, as TP53 CHIP is well-documented in elderly individuals. Accordingly, therapy-related pathogenesis should be considered plausible but not established in this case.

## Conclusion

This case report highlights the diagnostic challenge and management dilemma associated with a rare case of MCL in an 86-year-old patient with a history of DLBCL and prostate adenocarcinoma. This case demonstrates a therapy-related presentation (considered plausible given prior alkylating-agent exposure, though alternative explanations, including age-related CHIP, cannot be excluded), with suspected overlap with an associated myeloid neoplasm and highly complex genomic alterations (TP53, RB1, DAXX, ASXL1, TET2, SRSF2, and KIT p.D419del). The identification of a rare KIT p.D419del extracellular-domain mutation in the absence of detectable D816V highlights the importance of comprehensive NGS profiling and the use of sensitive assays to exclude low-VAF D816V, given its therapeutic implications. MCL is a rare disease with a very poor prognosis. This case serves as an important reminder for hospital providers to maintain a high index of suspicion for MCL among patients with persistent unexplained cytopenias and to pursue comprehensive genomic profiling to guide diagnosis, prognosis, and treatment planning. Expanding therapeutic options for MCL requires a deeper understanding of the molecular mechanisms involved and an evaluation of the feasibility of targeting them pharmacologically. Further research is needed to elucidate high-molecular-risk mutations in MCL and guide treatment strategies.

## Data Availability

All data supporting the findings of this study are available from the corresponding author upon reasonable request.
